# Myasthenia Gravis

**Published:** 1905-03-25

**Authors:** 


### Hospital Clinics.

### MYASTHENIA GRAVIS.

In a clinical lecture on this disease Dr. James Taylor[1] relates some personal experiences, and discusses the clinical and pathological aspects of the malady. The condition is characterised by great muscular weakness affecting for the most part the muscles which receive their nerve supply from the medulla oblongata; in addition the muscles of the limbs and trunk may also be affected, and this latter development may occasionally precede the weakness of the muscles supplied from the bulb. Ptosis is one of the earliest signs of myasthenia, and with it is frequently associated feebleness of some of the external ocular muscles. Hence the patient often complains of diplopia, the degree of this varying at different times in accordance with the degree of muscular exhaustion. Both the ptosis and the diplopia are found to be marked on some days, and practically absent on others. Nystagmoid movements may be present, but the pupil is rarely disturbed. Other usual evidences of muscular weakness are found in difficulty of mastication, so that the patient may acquire the habit of supporting the lower jaw with his hand whilst eating; weakness of the facial muscles, the patient as a result being unable to whistle or to blow out a light, and being troubled perhaps with dribbling of the saliva; inability to move the tongue freely; weakness of the soft palate giving rise to a nasal intonation; and difficulty in swallowing and articulation. All these facts are apt to be reduced to slight proportions by rest, and to be exaggerated by exertion. Hence they are for the most part more conspicuous in the later that the earlier part of the day. This feature of the disease—namely, the tendency for the muscular defect to appear under the influence of exertion—is well seen in connection with the act of articulation; at first the speech may be quite clear and the voice good, but after a little time a nasal tone may be detected and ultimately the patient becomes both voiceless and breathless, these conditions disappearing after a period of rest. Dyspnœa, in consequence of the ready exhaustion of the respiratory muscles, is easily provoked on exertion, and this constitutes a dangerous aspect of the disease as there may be actual crises of dyspnœa leading to a fatal result. Weakness of the upper limbs may be shown in various ways; one is a complaint on the part of female patients of inability to "do their back hair." In the lower limbs the quadriceps and the ilio-psoas suffer most; walking any considerable distance is usually quite impossible, and inability to ascend stairs or an incline is frequently noticed. Sometimes there are sudden falls from giving way of the muscles of the lower limbs. Emotional conditions, cold, and the menstrual period in women are circumstances which aggravate this condition of muscular weakness. The ready exhaustion of the muscles is well seen in the effects of faradism. At first the response may seem normal, but after repetition it becomes more and more feeble, and ultimately completely disappears. Similarly the tendon-jerks, which are usually very active, may in an advanced case be exhausted by repetition of the act of mechanical stimulation. Occasionally some degree of muscular atrophy may be appreciated, but in the majority of cases the bulk of the affected muscles retains its normal proportion. As regards etiology, males and females suffer in almost equal numbers; the age limits according to present records are 58 years at one extreme and eight years at the other, but probably further experience will extend these limits; most sufferers have been manual workers, but beyond this no specific occupation can be named in connection with the disease; mental strain, some acute illness, and over-exertion have each been regarded as an exciting cause of the disease; and occasionally the condition has developed upon some pre-existing weakness, as, for example, lead paralysis. In some instances several members of the same family have been affected.

So far as the nervous system is concerned, careful examination has failed to detect any histological change; and, save for an occasional slight degree of atrophy, the same is true of the affected muscles. The suggestion arising from the symptoms is that there is present in the blood some toxin which interferes with the function of the terminal part of the lower motor neurons. In this connection it is of interest to note that in some cases there has been present an enlarged thymus gland, and in others, tumours of lymphoid tissue have been found distributed through the muscles and other organs. Hence the suggestion naturally arises that from these sources possibly toxic material capable of prejudicing the motor terminations of the nerves may be contributed to the blood.

In the differential diagnosis, the disease, it must be remembered, closely resembles the condition due to gradual degeneration of the nuclei of the ocular muscles and of the muscles supplied from the bulb. Indeed, in a given case it may be impossible to say on a single examination whether the condition is myasthenia or the result of central organic disease. But the variability of the symptoms, the easy exhaustion of the muscles of the trunk and extremities, will indicate the existence of myasthenia. Diphtheritic paralysis is distinguished by the history of sore throat, by the absence of the knee-jerks as compared with their activity or excess in myasthenia, and by the rapid improvement under appropriate treatment. Hysteria has often been regarded as the explanation of the changeable and fluctuating character of the symptoms of myasthenia, but a careful examination will detect the weakness of the muscles, and especially of the ocular muscles, and will prevent the diagnosis of functional disease.

The prognosis in myasthenia is somewhat grave in view of the fact that there is danger of an affection of the respiratory motor apparatus, which may, even in the course of a few hours, lead to a fatal termination. On the other hand, in many patients the disease extends over a considerable number of years with little or no change, and in some cases recovery appears to have taken place. With rapid onset and progress the risk of fatal respiratory crises is to be feared, whilst a much more gradual development is not inconsistent with many years of life. The only remedy which has proved to be of value so far is strychnine given hypodermically; the suggestions already given in reference to the pathology of the disease would indicate that some antitoxin would be the rational treatment, but, unfortunately, no such agent is yet in our possession.

[1] Brit. Med. Jour, March 11, 1905, and the Polyclinic Jour, March, 1905.

